# DNA Ligase IV and Artemis Act Cooperatively to Suppress Homologous Recombination in Human Cells: Implications for DNA Double-Strand Break Repair

**DOI:** 10.1371/journal.pone.0072253

**Published:** 2013-08-14

**Authors:** Aya Kurosawa, Shinta Saito, Sairei So, Mitsumasa Hashimoto, Kuniyoshi Iwabuchi, Haruka Watabe, Noritaka Adachi

**Affiliations:** 1 Graduate School of Nanobioscience, Yokohama City University, Yokohama, Japan; 2 Department of Biochemistry, Kanazawa Medical University, Ishikawa, Japan; 3 Advanced Medical Research Center, Yokohama City University, Yokohama, Japan; Michigan State University, United States of America

## Abstract

Nonhomologous end-joining (NHEJ) and homologous recombination (HR) are two major pathways for repairing DNA double-strand breaks (DSBs); however, their respective roles in human somatic cells remain to be elucidated. Here we show using a series of human gene-knockout cell lines that NHEJ repairs nearly all of the topoisomerase II- and low-dose radiation-induced DNA damage, while it negatively affects survival of cells harbouring replication-associated DSBs. Intriguingly, we find that loss of DNA ligase IV, a critical NHEJ ligase, and Artemis, an NHEJ factor with endonuclease activity, independently contribute to increased resistance to replication-associated DSBs. We also show that loss of Artemis alleviates hypersensitivity of DNA ligase IV-null cells to low-dose radiation- and topoisomerase II-induced DSBs. Finally, we demonstrate that Artemis-null human cells display increased gene-targeting efficiencies, particularly in the absence of DNA ligase IV. Collectively, these data suggest that DNA ligase IV and Artemis act cooperatively to promote NHEJ, thereby suppressing HR. Our results point to the possibility that HR can only operate on accidental DSBs when NHEJ is missing or abortive, and Artemis may be involved in pathway switching from incomplete NHEJ to HR.

## Introduction

DNA double-strand breaks (DSBs) can be caused by exogenous and endogenous mechanisms, such as ionizing radiation, reactive oxygen species, or replication fork collapse [1]–[5]. Efficient repair of DSBs is thus crucial for cells to maintain genome integrity. Mammalian cells have evolved at least two distinct pathways for repairing DSBs, homologous recombination (HR) and nonhomologous end-joining (NHEJ) [6]. HR allows for accurate repair of DSBs with the use of homologous DNA sequences [2], [3], whereas NHEJ repairs broken DNA ends with little or no homology and is often associated with nucleotide loss [4], [5]. Because of such intrinsic differences in accuracy between HR and NHEJ, the two pathways should differentially contribute to repair of, and cellular survival after, different types of DSBs. Apparently, reliance on HR should be advantageous for cells to preserve genome integrity and indeed replication-associated DSBs appear to be preferentially repaired by HR [2], [3]. Unlike NHEJ, however, HR is only active when a sister chromatid is available, and NHEJ is typically a predominant pathway for DSB repair in mammalian cells. Thus, cells deficient in any of the NHEJ factors (Ku70, Ku80, DNA-PKcs, Artemis, XLF, XRCC4, and DNA ligase IV) show increased sensitivity to ionizing radiation [5], [7]. In human somatic cells, however, the respective roles of HR and NHEJ have yet to be elucidated. Additionally, we do not know yet how HR or NHEJ is chosen for repair in a cell, although it is generally assumed that HR and NHEJ (specifically, the Ku70/Ku80 heterodimer) compete for DSB ends on a first-come/first-“serve” basis [8]. More recent studies show that initiation of end-resection by CtIP and the MRN complex is one of the key mechanisms that influence the usage of HR and NHEJ upon DSBs [9]–[11].

Genetic analysis using gene-knockout mutants provides definitive tools to explore the respective roles of HR and NHEJ. Recently, we have developed an efficient gene-knockout system using the human pre-B cell line Nalm-6 [12], [13]. In this study, we employ this system to generate a series of human mutant cell lines that lack one or two genes involved in DSB repair; Rad54, DNA ligase IV and Artemis. Rad54 is a central HR protein that interacts with and stabilizes the Rad51 nucleoprotein filament [14], and is also involved in the dissociation of Rad51 from the filament [14]. Further, Rad54 can bind Holliday junction-like structures to promote their bidirectional branch migration in an ATPase-dependent manner [14]. Targeted disruption of the *Rad54* gene in mouse or avian cells results in a significant decrease in HR [15]–[17]. On the other hand, DNA ligase IV constitutes a ligase complex (i.e. the DNA ligase IV/XRCC4 complex) that is absolutely required for all NHEJ reactions to be completed, and other DNA ligases (I and III) cannot substitute for this critical function of DNA ligase IV [18]–[20]. Artemis is also involved in NHEJ, although its exact role remains elusive [4], [21]. Artemis is associated with and phosphorylated by DNA-PKcs, and acquires structure-specific endonuclease activity [4], [21]. We previously reported that Artemis-deficient cells exhibit increased sensitivity to low-dose, but not high-dose radiation [15], implying that Artemis has another role in DSB repair in addition to its role as an end-processing factor during NHEJ. Genetic analysis of those gene-knockout mutants not only allowed us to examine the respective roles of HR and NHEJ in the context of human somatic cells but also led us to suggest a novel concept for DSB repair, with a possible role of Artemis in pathway switching from uncompleted NHEJ to HR. Thus, this is the first report on genetic analysis of respective roles of NHEJ and HR in human cells and a novel role for Artemis in DSB repair.

## Results and Discussion

### Overlapping Roles of HR and NHEJ in Repairing Radiation-induced DSBs

To address the relative contribution of HR and NHEJ to DSB repair of human cells, we generated a series of knockout mutant cell lines deficient for DSB repair factors by using the Nalm-6 cell line, in which we have recently developed a system that enables rapid production of knockout mutants by gene targeting [12], [13]. Specifically, we knocked out the *RAD54* and *LIG4* genes to generate mutants deficient in HR or NHEJ, respectively (Figure S1 and [13]). We also generated a double-mutant deficient for both HR and NHEJ. Targeted gene disruption was verified by RT-PCR, Southern blot, or western blot analysis (Figure S1). Although the genetic deletion of *RAD54* or *LIG4* did not significantly affect cell growth or cell cycle distributions (Figure S2), the *LIG4* ^−/−^ *RAD54* ^−/−^ double-mutant grew more slowly than either single mutant, suggesting that HR and NHEJ have overlapping roles to maintain normal cell proliferation in human somatic cells.

**Figure 1:**
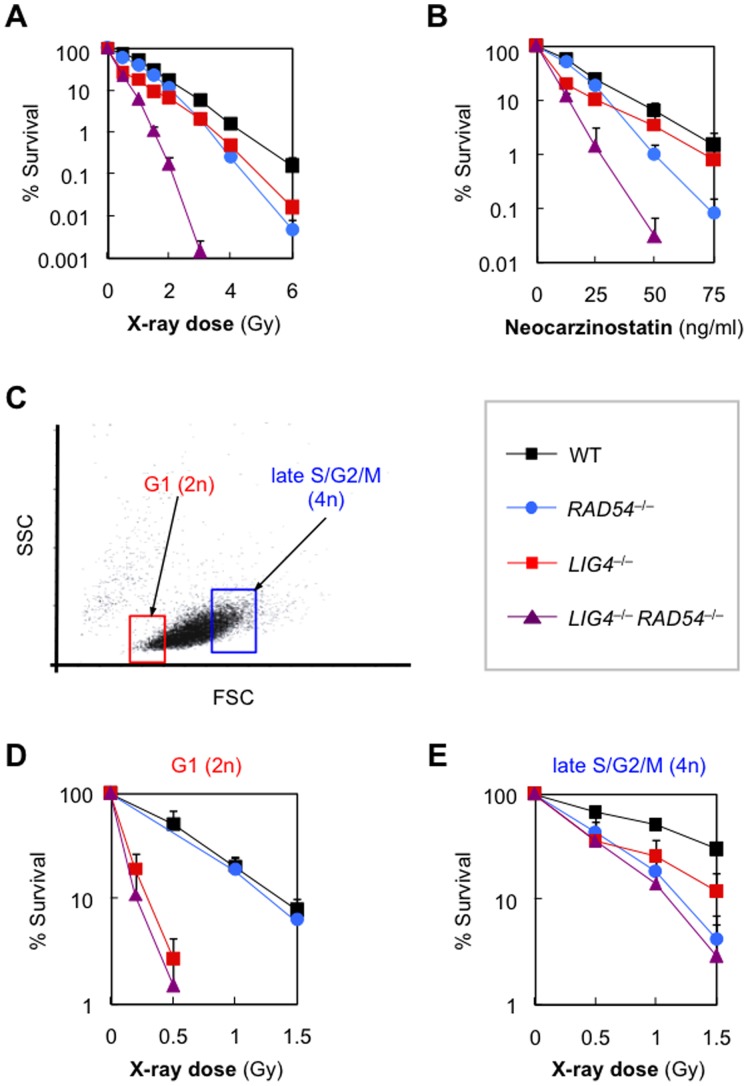
Overlapping roles of NHEJ and HR in repair of radiation-induced DSBs. (A, B) Sensitivity of *wild-type*, *RAD54* ^−/−^, *LIG4* ^−/−^, and *LIG4* ^−/−^ *RAD54* ^−/−^ cells to X-rays (A) or neocarzinostatin (B), as determined by clonogenic assays. Data are the mean ± SD of at least three independent experiments. Where absent, error bars fall within symbols. (C–E) Radiosensitivity of *wild-type*, *RAD54* ^−/−^, *LIG4* ^−/−^, and *LIG4* ^−/−^ *RAD54* ^−/−^ cells in G1- or late S/G2/M phase. Shown in (C) is a two-dimensional dot plot of FSC vs SSC. 2n- and 4n-cells were sorted using the indicated gates, and subjected to clonogenic assays (D, E).

To examine the sensitivity of the mutant cell lines to ionizing radiation, we performed clonogenic survival assays after X-irradiation. As shown in Figure 1A, *RAD54* ^−/−^ and *LIG4* ^−/−^ cells both displayed increased sensitivity to ionizing radiation. More specifically, *LIG4* ^−/−^ cells were more sensitive to low-dose radiation (<3 Gy) than were *RAD54* ^−/−^ cells, while the opposite was true for high-dose radiation. The requirement for NHEJ, rather than HR, in coping with low doses of radiation is consistent with the report using chicken DT40 cells [17]. In agreement with previous reports using mouse and chicken mutants [16], [17], the human *LIG4* ^−/−^ *RAD54* ^−/−^ double-mutant was more radiosensitive than either single mutant. Intriguingly, very similar results were obtained with neocarzinostatin, a radiomimetic agent that directly causes DSBs [22] (Figure 1B). We also sought to examine the relative contribution of HR and NHEJ during the cell cycle. For this, we fractionated 2n and 4n cells of each cell line, prior to X-irradiation, using a flow cytometric cell sorter [23](Figure 1C). We confirmed that at least 80% of fractionated 2n and 4n cells were in G1- or late S/G2/M-phase, respectively (data not shown). As shown in Figure 1D, *RAD54* ^−/−^2n cells exhibited almost the same radio-sensitivity as *wild-type* cells, while *LIG4* ^−/−^2n cells were highly radiosensitive, demonstrating the pivotal role of NHEJ in G1 phase upon accidental DSBs. In contrast, 4n-enriched *RAD54* ^−/−^ and *LIG4* ^−/−^ cells were similarly radiosensitive (Figure 1E), an observation consistent with the notion that NHEJ and HR are both important for repairing radiation-induced DSBs in post-replicated cells. Collectively, these results indicate that HR and NHEJ contribute independently to repair of radiation-induced DSBs in human cells.

### Absolute Requirement of DNA Ligase IV for Repair of Top2-induced DSBs

**Figure 2:**
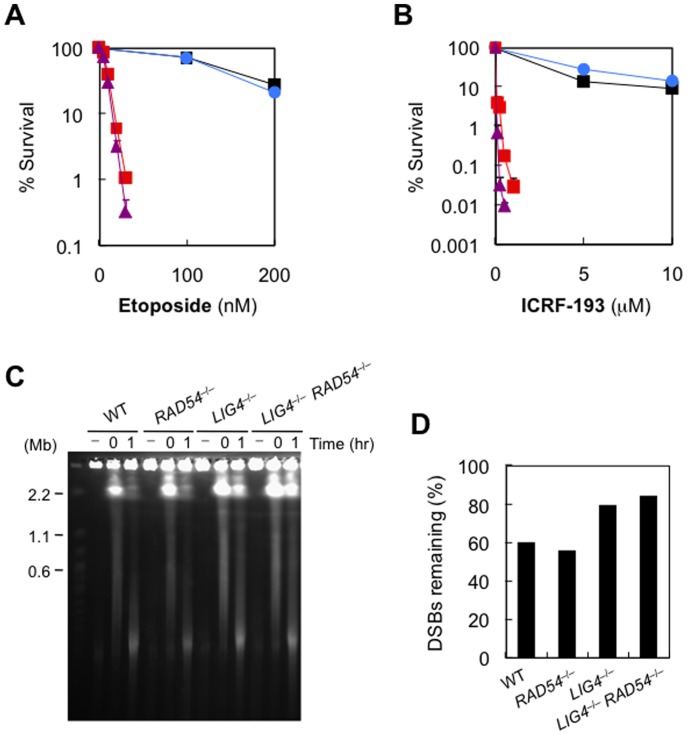
Absolute requirement of DNA ligase IV for repairing Top2-induced DSBs. (A, B) Sensitivity of *wild-type*, *RAD54* ^−/−^, *LIG4* ^−/−^, and *LIG4* ^−/−^ *RAD54* ^−/−^ cells to etoposide (A) and ICRF-193 (B), as determined by clonogenic assays. Data are the mean ± SD of at least three independent experiments. Where absent, error bars fall within symbols. Symbols are as in Figure 1. (C, D) PFGE analysis to detect unrepaired DSBs. Genomic DNA from untreated cells (–), cells treated with etoposide (100 µM) for 1 hr (0), or cells incubated in drug-free medium for 1 hr after treatment with etoposide (1) were subjected to PFGE (C), and DSBs after 1-hr incubation were quantified (D).

We next examined the sensitivity of the mutant cell lines to inhibitors of topoisomerase II (Top2), an enzyme that alters the topology of DNA [24]. Top2 inhibitors, such as etoposide or ICRF-193, have been shown to induce DSBs, by trapping Top2 cleavable complexes or closed clamps, respectively [24]–[26]. In yeast, HR plays central roles in the repair of Top2-mediated DNA damage, with NHEJ having no or little contribution [27]. As shown in Figure 2A and B, human *LIG4* ^−/−^ cells were extremely hypersensitive to etoposide and ICRF-193. In contrast, *RAD54* ^−/−^ cells showed only slightly increased sensitivity to etoposide, and no increased sensitivity to ICRF-193. These results indicate that NHEJ is absolutely required for repairing Top2-dependent DNA damage. We also performed PFGE analysis to confirm that etoposide-induced DNA damage was less efficiently repaired in *LIG4* ^−/−^ cells than in *wild-type* cells and *RAD54* ^−/−^ cells (Figure 2C and D). We note that *LIG4* ^−/−^ *RAD54* ^−/−^ cells were more, albeit slightly, sensitive to these drugs than were *LIG4* ^−/−^ cells, suggesting that HR also plays a role in repair, particularly when NHEJ is ablated. These observations were confirmed by using two independent clones of each genotype (Figure S3 and data not shown). In avian DT40 cells, absence of Rad54 partially alleviated hypersensitivity of NHEJ mutants to Top2 inhibitors [28]. This discrepancy may possibly reflect the extraordinarily high HR activity of DT40: in the presence of Top2 inhibitors, cellular processes, such as transcription, replication and presumably HR, are capable of converting Top2 proteins into irreversible cellular poisons. Thus, frequent HR reactions occurring in DT40 cells could have masked the positive contribution of HR to cell survival after Top2-induced DNA damage [28].

### Increased Resistance to Replication-dependent DSBs in Cells Lacking DNA Ligase IV

**Figure 3:**
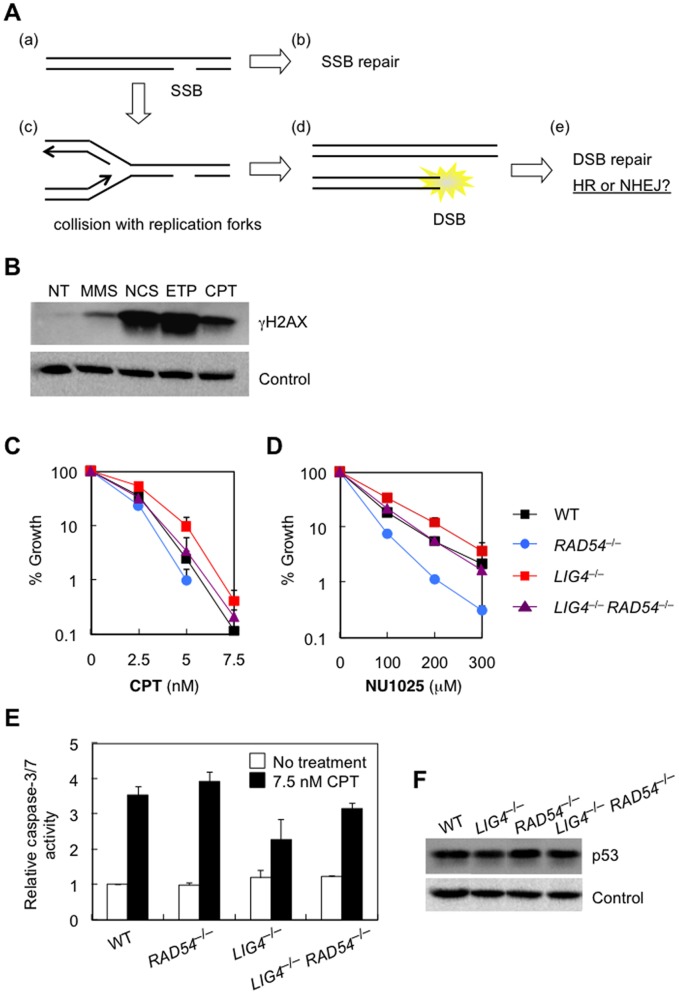
Genetic deletion of human DNA ligase IV confers resistance to killing by replication-associated DSBs. (A) Schematic representation of replication-associated DSBs. SSBs accumulate in the genome when cells are treated with CPT or NU1025 (a). In mammalian cells, SSBs are primarily repaired by the SSB repair pathway (b). If left unrepaired, however, SSBs are converted into DSBs, accompanied by fork collapse, upon collision with replication forks (c,d), and repaired by the DSB repair mechanism (e). (B) Detection of γ -H2AX using cells treated with MMS, neocarzinostatin, etoposide, or camptothecin as described in Materials and Methods. NT, untreated cells. (C, D) Sensitivity of *wild-type*, *RAD54* ^−/−^, *LIG4* ^−/−^, and *LIG4* ^−/−^ *RAD54* ^−/−^ cells to CPT (C) and NU1025 (D), as determined by growth inhibition assays. Data are the mean ± SD of at least three independent experiments. Where absent, error bars fall within symbols. (E) Relative caspase-3/7 activity after CPT treatment. (F) Western blot analysis for p53. Twenty micrograms of whole cell extract from wild-type (WT) and mutant cell lines were loaded on a 7.5% SDS-polyacrylamide gel.

The genotoxic agents used above are all capable of directly inducing DSBs, irrespective of cell cycle phase. Therefore, we next examined the sensitivity of our mutant human cell lines to DNA-damaging agents that can only induce DSBs in a replication-dependent fashion. For this purpose, we employed camptothecin (CPT) and NU1025. CPT is a potent inhibitor of topoisomerase I, which alters the topology of DNA through a transient single-strand break (SSB) and subsequent resealing of the nick [29]. Treatment of cells with CPT thus induces SSBs at each site at which the enzyme is covalently linked [29]. On the other hand, NU1025 is an inhibitor of poly(ADP-ribose) polymerase (PARP), which plays a central role in SSB repair [30]. The inhibition of PARP activity thus results in accumulation of unrepaired SSBs in the genome. Although these SSBs are primarily repaired by the SSB repair mechanism in mammalian cells, it is now well established that, in S phase, such SSBs caused by CPT or NU1025 are converted into DSBs, accompanied by fork collapse, upon collision with replication forks [31]–[33](Figure 3A). Indeed, we observed a substantial level of histone H2AX (Ser139) phosphorylation in CPT-treated cells (Figure 3B).

As shown in Figure 3C and D, *RAD54* ^−/−^ cells showed increased sensitivity to CPT and NU1025, suggesting that HR plays a role in repairing DSBs induced by these drugs. In sharp contrast, *LIG4* ^−/−^ cells showed no increased, rather decreased, sensitivity to these drugs. Intriguingly, the sensitivity of *RAD54* ^−/−^ cells was significantly alleviated by the deletion of *LIG4*, and, as a consequence, *LIG4* ^−/−^ *RAD54* ^−/−^ cells (deficient for HR and NHEJ) showed essentially the same sensitivity as *wild-type* cells (proficient for HR and NHEJ). Consistent with this, caspase-3/7 activity of *LIG4* ^−/−^ *RAD54* ^−/−^ cells after CPT treatment was significantly lower than that of *RAD54* ^−/−^ cells (Figure 3E). These results clearly show that NHEJ repair is detrimental to cells harboring replication-dependent accidental DSBs. We speculate that, unlike ‘direct DSBs’, replication-dependent DSBs have no correct DNA ends to be rejoined, so that repair by NHEJ results in a deleterious dead end structure of some sort, eventually leading to cell death [34]. We note that the increased resistance caused by *LIG4* deficiency has no direct relation to p53-dependent apoptosis, as *LIG4* ^−/−^ *TP53* ^−/−^ cells were more resistant to CPT than either single mutant (Figure S4A). In addition, caspase-3/7 activity of *LIG4* ^−/−^ *TP53* ^−/−^ cells after CPT treatment was even lower than that of *LIG4* ^−/−^ or *TP53* ^−/−^ cells (Figure S4B). Furthermore, the levels of p53 expression were essentially the same in *wild-type* and *LIG4* ^−/−^ mutant cell lines (Figure 3F). Together, these findings support the notion that the increased CPT resistance of *LIG4* ^−/−^ cells reflects the absence of toxic NHEJ events, rather than arrest or delay of cell cycle progression.

Importantly, our results suggest that upon accidental DSBs, cells are not necessarily choosing a suitable repair pathway for their survival (i.e. HR, in the case of CPT-induced damage; otherwise, *LIG4* ^−/−^ cells would have exhibited the same sensitivity as *wild-type* cells). Apparently, these findings do not conflict with the long-standing competition model [8], because absence of NHEJ should facilitate HR repair if the two pathways compete for DSB ends (and actually this was the case). However, along with the absolute requirement of NHEJ for Top2-mediated DNA damage and low-dose irradiation, one may favor another likely possibility. That is, NHEJ is chosen for most, or possibly all, accidental DSBs, and HR is primarily, or only, used when NHEJ is missing or NHEJ repair has failed (see below). This idea may be strongly supported by the fact that Ku, which triggers NHEJ reaction, is one of the most abundant proteins in mammalian cells (estimated at ∼4×10^5^ molecules per cell), with an equilibrium constant of ∼5×10^−10^ for DNA termini [5]. In this regard, it was reported that Ku80 is required for immobilization of DNA ends of broken chromosomes [35], [36]. Furthermore, live cell imaging techniques combined with laser micro-irradiation showed that Ku very quickly accumulates at the sites of DSBs [37]. Taken together, it may be that Ku can bind virtually all DSBs to promote NHEJ, possibly without competition. In other words, there may be a much stronger bias toward NHEJ than previously thought, even in the case of replication-associated DSBs that apparently rely on HR repair for cells to survive [38]–[40]. This should indeed be the case for cells in G1 phase, where HR repair cannot operate; therefore, it may be reasonable to speculate that cells are doing the same thing throughout the cell cycle.

### Generation of Artemis-knockout Human Cell Lines

Given the high NHEJ/HR ratio mentioned above, we reasoned that there might be a factor(s) that play a role in pathway switching from abortive NHEJ to HR. One such candidate is Artemis, which is a bona fide NHEJ factor that is physically associated with and phosphorylated by DNA-PKcs [21], [41] and also associates with the Mre11/Rad50/Nbs1 complex (involved in HR) in an ATM-dependent manner in response to radiation-induced DSBs [42]. Moreover, Cui *et al*. [43] have reported that the autophosphorylation status of DNA-PKcs may impact on DSB repair pathway choice in mammalian cells. These findings prompted us to generate human *ARTEMIS* ^−/−^ cells and *LIG4* ^−/−^ *ARTEMIS* ^−/−^ cells, which should give us clues to elucidate the role of Artemis in NHEJ and overall DSB repair. Targeted disruption of the human *ARTEMIS* gene was verified by Southern blot, RT-PCR and western blot analysis, allowing us to isolate two *ARTEMIS* ^−/−^ cell lines [15] and three *LIG4* ^−/−^ *ARTEMIS* ^−/−^ cell lines (Figure S5 and data not shown). Expression of other NHEJ factors (DNA-PKcs, Ku70 and Ku80) was unaffected by the *ARTEMIS* deletion, as confirmed by western blot analysis (Figure S5 and data not shown). Consistent with our previous report [15], genetic deletion of *ARTEMIS*, unlike *LIG4* deletion, significantly affected cell growth, though flow cytometric analysis of the mutant cell lines revealed no significant difference in the cell cycle distributions of asynchronous cells (Figure S6). The reduced growth rate of *ARTEMIS* ^−/−^ cells was not further affected by loss of DNA ligase IV. These results suggest that besides its role as a core NHEJ protein, Artemis has another cellular function that is nonepistatic with DNA ligase IV function.

### Loss of Artemis Alleviates Hypersensitivity of Ligase IV-null Mutant to Ionizing Radiation and Top2-induced DSBs

**Figure 4:**
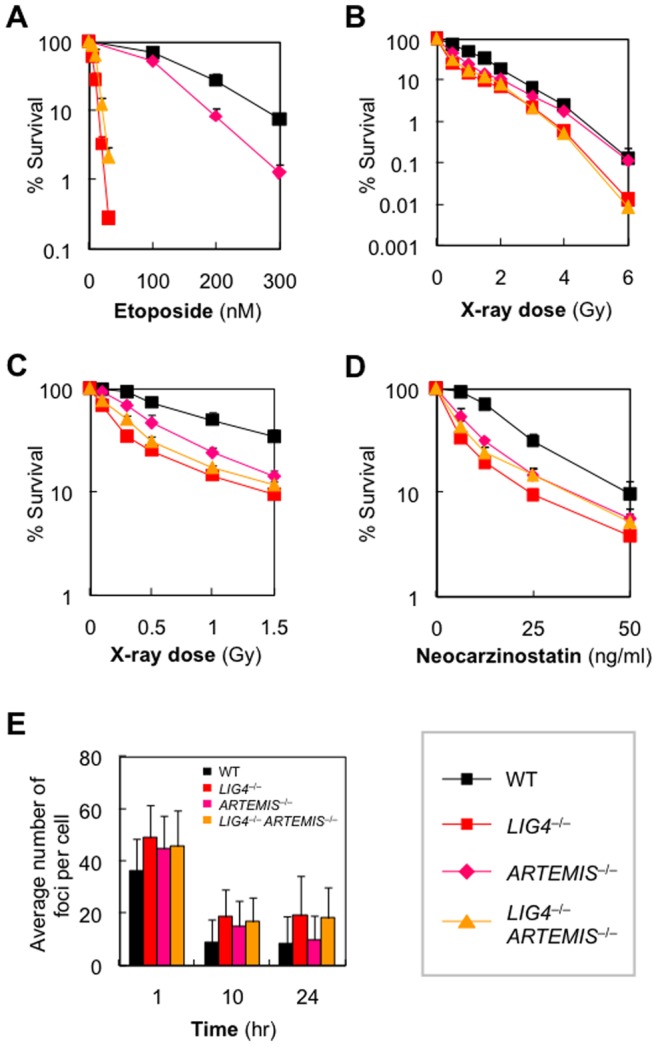
Loss of Artemis alleviates hypersensitivity of *LIG4* ^−/−^ cells to low-dose irradiation- and Top2-induced DSBs. (A–D) Sensitivity of *wild-type*, *ARTEMIS* ^−/−^, *LIG4* ^−/−^, and *LIG4* ^−/−^ *ARTEMIS* ^−/−^ cells to etoposide (A), X-rays (B and, for low-dose range, C), and neocarzinostatin (D), as determined by clonogenic assays. Shown are the mean ± SD of at least three independent experiments. Where absent, error bars fall within symbols. (E) Average number of γ-H2AX foci per cell. γ-H2AX focus-formation assay was performed using 1 Gy-irradiated cells.

The DNA damage that most absolutely requires NHEJ is that induced by Top2 inhibitors (Figure 2A). To gain further insight into the function of Artemis in DSB repair, we next examined etoposide sensitivity of Artemis-deficient cell lines. Consistent with a limited role of Artemis as an NHEJ protein [15], *ARTEMIS* ^−/−^ cells exhibited increased etoposide sensitivity, though to a much lesser extent than *LIG4* ^−/−^ cells (Figure 4A). Surprisingly, however, the hypersensitivity of *LIG4* ^−/−^ cells was alleviated by *ARTEMIS* inactivation (Figure 4A), and this observation was confirmed using three independent *LIG4* ^−/−^ *ARTEMIS* ^−/−^ clones (Figure S6C). Additionally, we examined the sensitivity to other types of Top2 inhibitor and obtained essentially the same results (data not shown). These data suggest that in the absence of DNA ligase IV, Artemis acts negatively for cell survival upon DSBs accidentally occurred in the genome, and thus loss of Artemis facilitates repair of those DSBs by other pathway(s) (most presumably, HR) when cells need NHEJ yet the critical ligase is missing. If so, *LIG4* ^−/−^ *ARTEMIS* ^−/−^ cells should similarly display milder sensitivity than *LIG4* ^−/−^ cells to low-dose X-rays, which require NHEJ rather than HR. This was indeed the case: cells lacking both Artemis and DNA ligase IV were more resistant to low-dose X-rays (≤2 Gy) than were *LIG4* ^−/−^ cells (Figure 4B and C). Moreover, similar results were obtained using neocarzinostatin (Figure 4D); in this case, *LIG4* ^−/−^ *ARTEMIS* ^−/−^ cells showed almost the same sensitivity as did *LIG4* ^−/−^ cells. This may possibly reflect the fact that HR repairs neocarzinostatin-induced DNA damage (cf. Figure 1A versus 1B). Using 1 Gy-irradiated cells, we counted γ-H2AX foci and found that the number of γ-H2AX foci correlated inversely with the survival rate of irradiated cells; in particular, the mean number of γ-H2AX foci per nucleus was slightly lower in *LIG4* ^−/−^ *ARTEMIS* ^−/−^ cells than in *LIG4* ^−/−^ cells (Figure 4E). Collectively, our results unequivocally suggest that, although Artemis is indeed involved in NHEJ, the protein acts negatively for cell survival when DNA ligase IV is not available.

### Loss of Artemis Leads to Increased Resistance to Replication-dependent DSBs Independently of DNA Ligase IV

**Figure 5:**
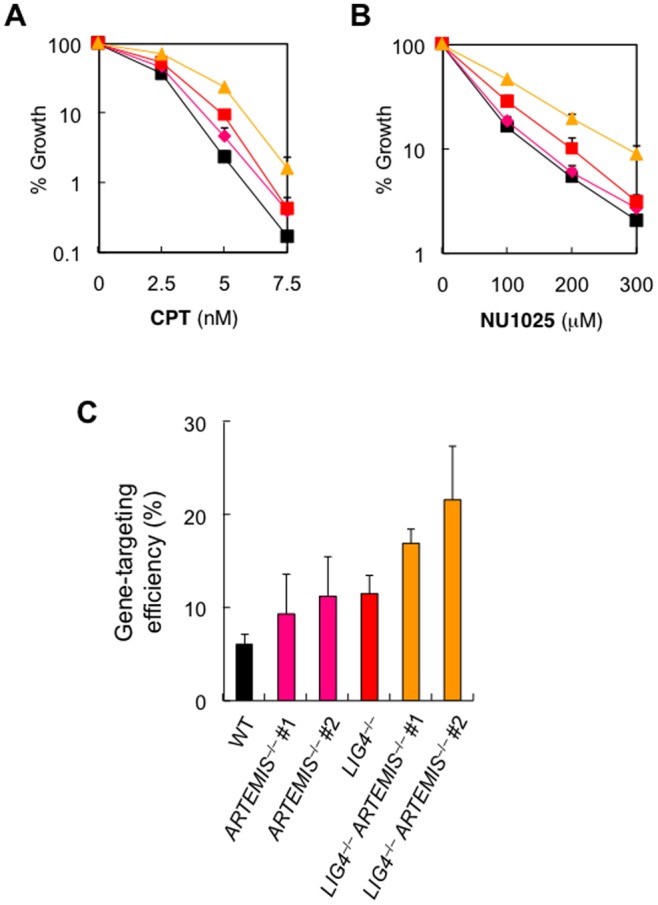
DNA ligase IV and Artemis deletion independently contribute to increased cellular resistance to replication-induced DSBs and increased gene targeting. (A, B) Sensitivity of *wild-type*, *ARTEMIS* ^−/−^, *LIG4* ^−/−^, and *LIG4* ^−/−^ *ARTEMIS* ^−/−^ cells to CPT (A) and NU1025 (B), as determined by growth inhibition assays. Symbols are as in Figure 4. Data are the mean ± SD of three independent experiments. Where absent, error bars fall within symbols. (C) Gene-targeting efficiency of *wild-type*, *ARTEMIS* ^−/−^, *LIG4* ^−/−^, and *LIG4* ^−/−^ *ARTEMIS* ^−/−^ cells. The gene-targeting efficiency at the *HPRT* locus was determined by the ratio of the number of targeted clones to that of clones analyzed.

Assuming that loss of Artemis facilitates HR repair in a cell, a likely function of Artemis would be to keep the ongoing NHEJ reaction active (irrespective of the presence/absence of DNA ligase IV), possibly thereby preventing HR from participating in the repair. In such a scenario, cells lacking both Artemis and DNA ligase IV would be more resistant than either single mutant toward replication-dependent DNA damage. Again, this was indeed the case: similar to *LIG4* ^−/−^ cells, *ARTEMIS* ^−/−^ cells showed increased resistance to CPT and NU1025, and, notably, *LIG4* ^−/−^ *ARTEMIS* ^−/−^ cells were even more resistant to CPT and NU1025 than either single mutant (Figure 5A and B). These results indicate that, similar to DNA ligase IV, Artemis is unfavorable for survival of cells harboring accidentally caused replication-associated DNA damage and, most importantly, Artemis and DNA ligase IV deletion contribute independently to the increased resistance to replication-associated DNA damage.

### Loss of Artemis Leads to Increased Gene Targeting Efficiencies

To directly test the idea that Artemis may serve to repress HR, we examined the impact of Artemis deficiency on the efficiency of gene targeting. For this purpose, we employed a targeting vector for the *HPRT* locus, the disruption of which confers 6-thioguanine-resistance to cells, thereby enabling rapid detection of gene-targeting events [44]. As shown in Figure 5C, *ARTEMIS* ^−/−^ cells consistently displayed higher gene-targeting efficiencies than did Artemis-proficient *wild-type* cells, and this enhancement was even more evident in the absence of DNA ligase IV. We note that the frequency of random integration was unaffected by the Artemis deficiency (data not shown). These data provide direct evidence that loss of Artemis and DNA ligase IV contribute independently to promoting HR in human somatic cells.

### NHEJ: the First Choice upon Accidental DSBs in Human Cells

**Figure 6:**
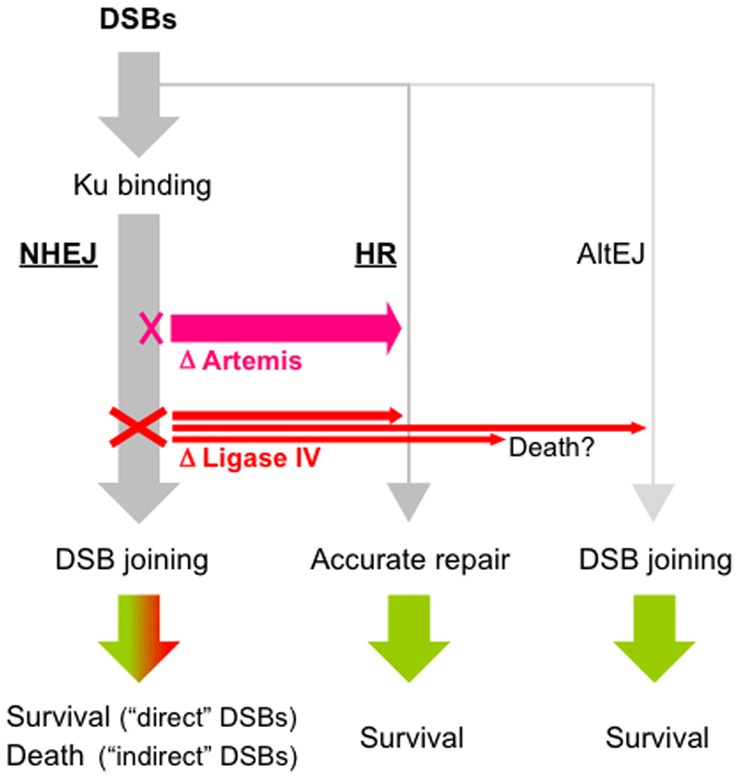
Model for DSB repair control in human somatic cells. Cells can suffer various types of DSBs, involving those induced by irradiation, Top2 (“direct DSBs”) and, in S phase, replication fork collapse (“indirect” DSBs). The Ku70/Ku80 complex, Ku, can rapidly bind to most, if not all, DSBs to initiate an NHEJ reaction. Ku-unbound DSBs, if any, are not subjected to NHEJ, but can be repaired by HR or alternative end-joining (AltEJ) pathways [58]–[60]. Irrespective of accuracy, those repair events lead to cellular survival, with the exception that NHEJ after indirect DSBs is toxic and leads to cell death. Loss of DNA ligase IV completely abolishes NHEJ and shunts the DSB toward HR or AltEJ, or may result in cell death [18]. Loss of Artemis, on the other hand, does not completely block the NHEJ reaction, but would more efficiently shunt the DSB to HR. Similar situations (i.e., some Ku-bound DSBs are not mended by NHEJ) can be caused by incomplete end-trimming reactions [61] and/or when the cell has a huge number of DSBs. When NHEJ is unsuccessful at rejoining the DSB, the cell would give up the abortive NHEJ reaction by somehow relieving the Artemis-mediated HR suppression. See text and Figure S7 for further details.

The observations described herein altogether support the idea of the NHEJ/HR competition model, but with a much stronger bias toward NHEJ than previously considered, as depicted in Figure 6. In this model, upon any types of DSBs, cells may first choose NHEJ for repair by virtue of efficient Ku binding to the ends. Importantly, this model well explains the high NHEJ/HR ratio and should be applicable to most, if not all, types of DSBs. Even more intriguingly, our data suggest that loss of Artemis efficiently shifts the balance toward HR. A more detailed model for DSB repair control is illustrated in Figure S7.

Notably, we observed additive effects of mutations of two NHEJ factors, Artemis and DNA ligase IV, on cellular tolerance to replication-associated DNA damage. This is likely due to the prevention of toxic NHEJ events after such damage, as well as a more efficient shift from incomplete NHEJ to HR. One possible scenario is that loss of DNA ligase IV completely prevents toxic NHEJ but may not allow for a shift from abortive NHEJ to HR. Loss of Artemis, on the other hand, only partially prevents toxic NHEJ, but may allow for an efficient shift from abortive NHEJ to HR. Alternatively, it is possible that Artemis may have a more direct role to prevent HR from participating in repair. It is also interesting to note that increased HR activity in the absence of Artemis may explain why Artemis-null cells do not show hypersensitivity to high-dose radiation (Figure 4B).

Several reports suggest that Artemis is phosphorylated by ATM as well as DNA-PKcs [42], [45], [46]. Löbrich and Jeggo [47] reported that ATM is required for Artemis function in response to ionizing radiation, but not in V(D)J recombination. Possibly, ATM may cooperate with Artemis to function in NHEJ (presumably, end-trimming reactions) and/or the “switching” in DSB repair. In this regard, it is interesting to note that the lethal phenotype of *Lig4* knockout mice is partially rescued by *Atm* deletion [48]. ATM is required for the full activation of DNA-PK and subsequent DSB repair [49], while Ku is reported to modulate ATM (and ATR) signaling pathways in response to DSBs [50]. It thus appears that multiple DNA repair proteins cooperate to regulate DSB repair, and further work is required to elucidate the precise mechanism for DSB repair switching, particularly in light of Artemis functions described herein. In this respect, it will be interesting to knock out the *ATM* and *DNA-PKcs* genes in Nalm-6 and its Artemis-deficient cell lines.

Apparently, the use of Ku-knockout cells should be valuable for further analysis of DSB repair mechanisms. Indeed, mutations of Ku and DNA ligase IV have different outcomes in mouse and chicken [18], [20]. It is important to note, however, that *KU70/KU80* are most likely essential genes in human somatic cells [51]–[53]. In fact, despite efforts to isolate Ku70-null Nalm-6 cells, we have been unable to disrupt the second *KU70* allele in *KU70* ^+/−^ mutant (our unpublished observations). The differential roles of Ku proteins between human and non-human cells highlight the importance and validity of genetic analysis using human somatic cells.

In the yeast *Saccharomyces cerevisiae*, HR repairs virtually all DSBs, yet NHEJ does have a role in DSB repair. In this organism, the competition (first-come/first-“serve”) model does stand; namely, Rad52 initiates HR, while Ku does NHEJ [8]. By contrast, in mammals, it has been a key issue to solve how cells choose a proper repair pathway when DSBs arise [40], [54]. Our results presented here clearly show that cells are not choosing a proper pathway for repairing DSBs. It could be that the competition model is applicable to higher eukaryotes as well, but clearly vertebrate Rad52 does not possess a similar function to yeast Rad52 [55]. Rather, the overwhelming abundance and high affinity to DNA termini of Ku proteins may support the concept of a stronger bias for NHEJ than previously appreciated. We speculate that the predominance of Ku-initiated NHEJ repair is advantageous for cells to maintain overall genome integrity, by facilitating rapid DSB repair throughout the cell cycle as well as by suppressing unfavorable HR events that could lead to gross chromosomal rearrangements.

### Conclusion

In this paper, we have conducted genetic analysis of DSB repair control by using a series of human gene-knockout cell lines. Our results clearly suggest that, upon accidental DSBs, NHEJ is the highly predominant repair pathway in human somatic cells, while HR may only become active when an NHEJ reaction has failed. We have also shown that Artemis promotes NHEJ independently of the critical NHEJ ligase, and indeed cells doubly deficient for Artemis and DNA ligase IV display an increased efficiency of gene targeting. Finally, the dominance of NHEJ over HR well explains the fact that an HR defect only affects high-dose irradiated cells where NHEJ is unable to deal with all the DSBs present.

## Materials and Methods

### Cell Culture and Transfection

The human pre-B cell line Nalm-6 and its derivatives were cultured at 37°C in ES medium (Nissui Seiyaku Co., Tokyo, Japan) supplemented with 10% calf serum (Hyclone, Logan, UT) and 50 µM 2-mercaptoethanol. The *LIG4^−/−^* and *ARTEMIS^−/−^* cells were generated as described [13], [15]. DNA transfection was performed as described previously [15]. Briefly, 4×10^6^ cells were electroporated with 4 µg of linearized targeting construct, cultured for 22 hr, and replated at a density of 0.5–1×10^6^ cells per 90-mm dish into agarose medium containing 0.5 µg/ml puromycin or 0.4 mg/ml hygromycin B (Wako Pure Chemical, Osaka, Japan). Alternatively, cells were diluted and divided into 96-well multiwell plates, so that each well contains 5×10^3^ cells per 0.2 ml of growth medium containing 0.4 mg/ml hygromycin B. After a 2–3 week incubation, genomic DNA was prepared from drug-resistant colonies and subjected to PCR and Southern blot analysis as described [15].

### DNA-damaging Agents

Neocarzinostatin, methyl methanesulfonate and camptothecin were purchased from Sigma-Aldrich (St. Louis, MO). Etoposide was purchased from BioVision (Mountain View, CA), NU1025 from Calbiochem (San Diego, CA), and ICRF-193 from Zenyaku Kogyo (Tokyo, Japan).

### Targeting Constructs

*RAD54* targeting constructs were designed to replace exons 4 to 7 with a floxed puromycin or hygromycin resistance gene. Briefly, 2.5- and 3.6-kb *RAD54* fragments were PCR amplified using Nalm-6 genomic DNA as template with primers R54-1 (5′-GGGGACAACTTTGTATAGAAAAGTTGCACATTCTTCCTTACCAGTTATGC-3′) and R54-2 (5′-GGGGACTGCTTTTTTGTACAAACTTGTACCACAGACTTAGCCAACCTGAG-3′) for the 5′-arm, and R54-3 (5′-GGGGACAGCTTTCTTGTACAAAGTGGGCCAGAGTCCAGAGTGCAAGCCAG-3′) and R54-4 (5′-GGGGACAACTTTGTATAATAAAGTTGAAAAGCGTTACTGGGAGGAAGATG-3′) for the 3′-arm. The MultiSite Gateway system (Life Technologies, Carlsbad, CA) was employed to assemble two genomic fragments and a drug resistance gene cassette, as described [13].

*ARTEMIS* targeting constructs were made as previously described [15]. Briefly, the targeting vector Artemis-Puro was designated to replace exons 8 and 9 with a floxed puromycin resistance gene. Likewise, the Artemis-Hyg vector was designated to replace exons 6 to 9 with the hygromycin resistance gene.

### Western Blot Analysis

Western blot analysis was performed as previously described [15]. The antibodies used in this study were anti-Artemis antibody (PAB-10241, Orbigen, San Diego, CA), anti-Ku70 antibody (K91620, BD Biosciences, San Diego, CA), anti-Ku80 antibody (K92620, BD Pharmingen), anti-γ-H2AX antibody (JBW301, Merck, Billerica, MA), anti-p53 antibody (DO-1, Santa Cruz Biotechnology, Santa Cruz, CA), and anti-DNA ligase IV antibody (a gift from H. Teraoka, Tokyo Medical and Dental University). To detect γ-H2AX, 5×10^5^ cells were treated with 200 mM methyl methanesulfonate, 100 ng/ml neocarzinostatin, 500 nM etoposide, or 10 nM camptothecin for 1 hr, washed twice with pre-chilled PBS containing 20 mM NaF and 10 mM Na_3_VO_4_, and lysed with 100 µl of lysis buffer (50 mM Tris-HCl (pH 7.4), 150 mM NaCl, 0.3% NP-40, 1% Tween-20, 20 mM NaF, 10 mM Na_3_VO_4_, and protease inhibitor cocktail (Sigma-Aldrich)).

### RT-PCR

Total RNA was extracted from each cell line using TRIzol reagent (Life Technologies), according to the manufacture’s instructions. Two micrograms of total RNA were reverse-transcribed by M-MLV reverse transcriptase (Promega, Madison, WI) according to the manufacture’s protocol using Oligo(dT)_15_ primer. The reaction mixture was incubated for 1 hr at 42°C, followed by PCR using the following primers: 5′-TGGCTCATGGGTACTTGACG-3′ and 5′-GACACCAGCACTACTTTGTC-3′ for *RAD54*, and 5′-CTTGTCATCAATGGAAATCC-3′ and 5′-GATGTCATCATATTTGGCAG-3′ for *GAPDH*.

### Flow Cytometric Analysis

Flow cytometric analysis was performed as described previously [15].

### Sensitivity Assays

Clonogenic assays were performed as described previously [15]. Briefly, 1×10^2^–2×10^5^ cells were plated into 60-mm dishes containing 5 ml of agarose medium with various concentrations of DNA-damaging agents. For X-ray sensitivity assays, cells were plated as above and exposed to various doses of X-ray using an X-ray generator (MBR-1520R, Hitachi Power Solutions, Ibaraki, Japan). Cell cycle-dependent X-ray sensitivity assay was performed as described previously [23]. Briefly, exponentially growing cells were applied to a JSAN cell sorter (Bay Bioscience, Hyogo, Japan) and sorted into groups based on the different stages of the cell cycle at room temperature over a 90-min period for the cells. All measurements were made using an argon laser turned at 488 nm. The sorted cells were washed once with growth medium, suspended in growth medium, and then subjected to clonogenic assay.

For growth inhibition assays, 2×10^4^ cells were seeded into 24-well plates and cultured for 96 hr in growth medium containing various concentrations of DNA-damaging agents. Cell proliferation was then measured using the CellTiter-Glo Luminescent Viability Assay kit (Promega). At least three independent experiments were performed for each assay.

For apoptosis analysis, 2×10^4^ cells were seeded into 24-well plates and cultured for 24 hr in growth medium with or without 7.5 nM CPT. Caspase-3/7 activity was then measured using the Caspase-Glo 3/7 Assay kit (Promega).

### Pulsed-field Gel Electrophoresis (PFGE) Analysis

PFGE assay was performed as described previously [56]. Briefly, cells were treated with or without 100 µM etoposide for 1 hr, washed once with PBS, and cultured for 1 hr. Chromosome-sized DNA was prepared from the cells using the CHEF Genomic DNA Plug Kit (Bio-Rad, Hercules, CA) and subjected to PFGE. DSBs were quantified using a MultiGauge software (Fuji Film Co., Tokyo, Japan).

### γ-H2AX Focus-formation Assay

γ-H2AX focus-formation assay was performed as described previously [57]. Briefly, cells were irradiated with X-rays, and cultured for 1, 10 or 24 hr. Cells were then attached to the surface of a slide glass by centrifugation, fixed with 4% paraformaldehyde for 10 min, permeabilized in PBS containing 0.3% NP-40, blocked in 3% bovine serum albumin at room temperature for 20 min, and then incubated with the anti-γ-H2AX antibody JBW301 at room temperature for 1 hr. After extensive washing, cells were incubated with fluorescein isothiocyanate-conjugated goat anti-mouse IgG antibody (Life Technologies) at room temperature for 1 hr. Cells were counterstained with 4′,6′-diamino-2-phenylindole, and foci of nonapoptotic nuclei were counted using an all-in-one type fluorescence microscope BZ-8000 (Keyence, Osaka, Japan).

### Gene Targeting Assay

Gene-targeting assay was performed using the *HPRT* locus (located on the X chromosome) essentially as described [44]. Briefly, cells were transfected with a linearized targeting vector, pHPRT-Hyg [44], and resulting hygromycin-resistant clones were transferred into growth medium containing 20 µM 6-thioguanine (Sigma-Aldrich). Correct targeting events were further confirmed by genomic PCR using primers HPRT-F (5′-TGAGGGCAAAGGATGTGTTACGTG-3′) and HPRT-R (5′-TTGATGTAATCCAGCAGGTCAGCA-3′).

## Supporting Information

Figure S1 **Generation of** ***RAD54*** **^−/−^ and** ***LIG4*** **^−/−^** ***RAD54*** **^−/−^ cells.** (A) Schematic representation of targeted disruption of the human *RAD54* gene. Th*e RAD54* locus, two targeting constructs (RAD54-Puro and RAD54-Hyg), and targeted locus are shown. Gene-targeting events replace exons 4 to 6 with the puromycin resistance gene (Puro^r^) or hygromycin resistance gene (Hyg^r^), flanked by *lox*P sequences. The black boxes and triangles represent exons and *lox*P sequences, respectively. The figure is not drawn to scale. (B) Southern blot analysis. EcoRI-digested genomic DNA of wild-type (+/+), heterozygous mutant (+/−), and homozygous mutant (−/−) cells was hybridized with the probe shown in (A). (C) RT-PCR analysis. Total cellular RNA was isolated from wild-type (WT), *LIG4* ^−/−^, *RAD54* ^−/−^, *LIG4* ^−/−^ *RAD54* ^−/−^, *ARTEMIS* ^−/−^, and *LIG4* ^−/−^ *ARTEMIS* ^−/−^ cells, and used as template to amplify cDNA for *RAD54* and *GAPDH*.
(TIF)

Figure S2 **Growth properties of wild-type and mutant cell lines.** (A) Growth curves of wild-type (WT), *RAD54* ^−/−^, *LIG4* ^−/−^, and *LIG4* ^−/−^ *RAD54* ^−/−^ cells. Shown are the mean ± SD of four independent experiments. (B) Percentage of cells in G1, S, G2/M, and subG1. Shown are the mean ± SD of three independent experiments.
(TIF)

Figure S3 **Absolute requirement of NHEJ in repair of etoposide-induced DNA damage.** Etoposide sensitivity of various mutant cell lines was determined by clonogenic assays. Data are the mean ± SD of at least three independent experiments. Where absent, error bars fall within symbols.
(TIF)

Figure S4 **Increased resistance of** ***LIG4*** **^−/−^ cells to CPT is unrelated to p53 function.** Shown is the sensitivity to CPT of wild-type (WT), *LIG4* ^−/−^, *TP53* ^−/−^, and *LIG4* ^−/−^ *TP53* ^−/−^ cells, as determined by growth inhibition assays.
(TIF)

Figure S5 **Generation of** ***ARTEMIS^−/−^*** **and** ***LIG4*** **^−/−^** ***ARTEMIS*** **^−/−^ cells.** (A) Scheme for 1st gene targeting. The human *ARTEMIS* gene (also known as *SCIDA*, *SNM1C*, or *DCLREC1C*) is composed of 14 exons, located on chromosome 10p13 (http://www.cgal.icnet.uk/DNA_Repair_Genes.html). The targeting vector Artemis-Puro was designed to replace exons 8 and 9 with the puromycin resistance (Puro^r^) gene. Triangles represent *lox*P sequences. (B) Scheme for 2nd gene targeting. The targeting vector Artemis-Hyg was designed to replace exons 6 to 9 with the hygromycin resistance (Hyg^r^) gene. Symbols are as in (A). (C) Western blot analysis for Artemis, Ku70, Ku80 and DNA ligase IV. Twenty micrograms of whole cell extract from wild-type (WT) and mutant cell lines were loaded on a 7.5% SDS-polyacrylamide gel.
(TIF)

Figure S6 **Growth properties of wild-type and mutant cell lines.** (A) Growth curves of wild-type (WT), *ARTEMIS* ^−/−^, *LIG4* ^−/−^, and *LIG4* ^−/−^ *ARTEMIS* ^−/−^ cells. Shown are the mean ± SD of three independent experiments. (B) Percentage of cells in G1, S, G2/M, and subG1. Shown are the mean ± SD of three independent experiments.
(TIF)

Figure S7 **Model for DSB repair control in human somatic cells.** Cells can suffer various types of DSBs, involving those induced by irradiation, Top2 and, in S phase, replication fork collapse (*i*). In this model, the Ku70/Ku80 complex can rapidly bind to most, if not all, DSBs to initiate an NHEJ reaction (*ii-a*). Ku-unbound DSBs, if any, are not repaired by NHEJ, but by HR (*ii-b*). After the Ku binding, the DNA ligase IV/XRCC4 complex rejoins the DSB when end-trimming is unnecessary (this would only be true for “clean” ends; e.g., signal joint formation during V(D)J recombination [5]) (*iii*). In most cases, end-trimming is required prior to rejoining, so that DNA-PKcs, Artemis, and DNA polymerases are recruited to the DSB to trim the ends (*iv*) [5]. After the trimming, the DSB can be rejoined by the DNA ligase IV/XRCC4 complex (*v-a*), in the absence of which, however, the break remains unrejoined [18]. Such situations can be caused by incomplete end-trimming reactions [61] and/or when the cell has a very large number of DSBs. (Regarding the latter, it is particularly interesting to note that the expression level of DNA ligase IV (and XRCC4) is considerably lower than Ku70/Ku80 [12]; thus, it is reasonable to speculate that all the Ku-bound DSBs cannot be rejoined by DNA ligase IV.) In these cases NHEJ may repeatedly perform end-trimming and ligation reactions (*v-b*). It could be that the presence of Artemis may assure these reactions; namely, Artemis may serve to suppress switching from the incomplete NHEJ reaction to HR (*vi*), though such unrejoined DSBs may cause cell death (*v-c*). When NHEJ is unsuccessful at rejoining the DSB, the cell gives up the abortive NHEJ reaction by somehow relieving the Artemis-mediated HR suppression. Then, HR finally gets the opportunity to repair the DSB (*vii*). (Possibly, DNA-PKcs may change its autophosphorylation status to facilitate HR [62].) Alternatively, or additionally, those DSBs that remain unrejoined may be shunted to an alternative end-joining (EJ) pathway [58]–[60] or may result in cell death [18] (*viii*).
(TIF)
