# Supplementary figures and images for: DNA Ligase IV and Artemis Act Cooperatively to Suppress Homologous Recombination in Human Cells: Implications for DNA Double-Strand Break Repair
Source: PLoS One. 2013 Aug 14;8(8):e72253. doi: 10.1371/journal.pone.0072253 (PMC3743779; doi:10.1371/journal.pone.0072253)

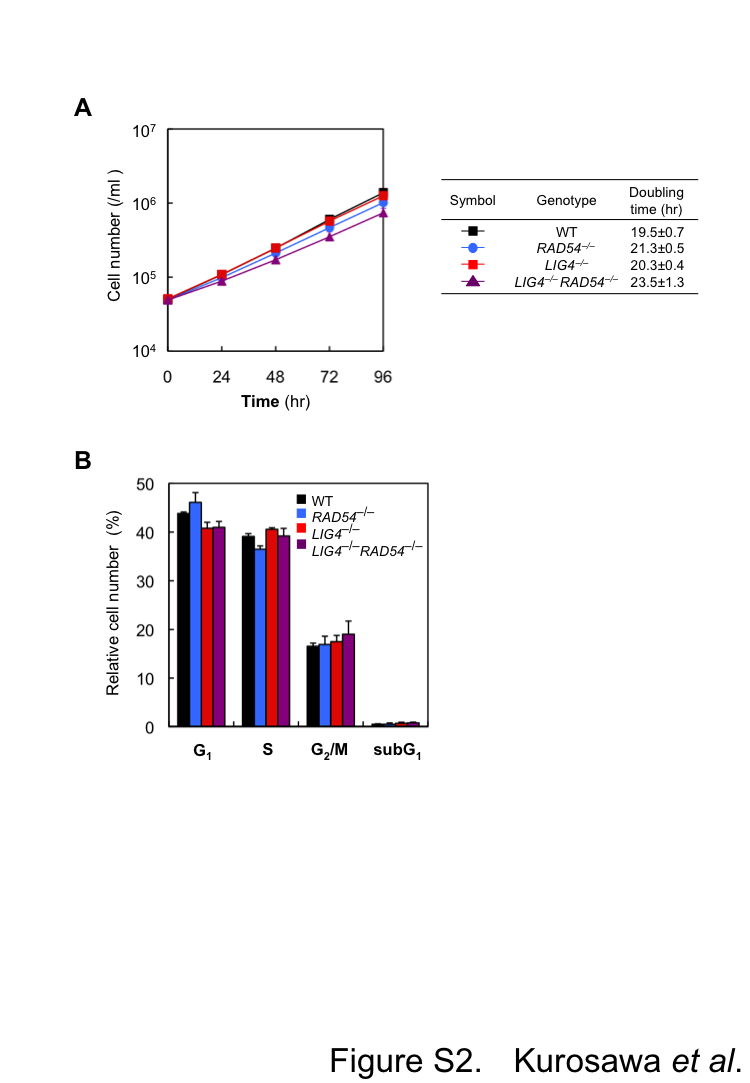

Supplement: Figure S2 — Growth properties of wild-type and mutant cell lines. (A) Growth curves of wild-type (WT), RAD54 −/−, LIG4 −/−, and LIG4 −/− RAD54 −/− cells. Shown are the mean ± SD of four independent experiments. (B) Percentage of cells in G1, S, G2/M, and subG1. Shown are the mean ± SD of three independent experiments. (TIF) [file pone.0072253.s002.tif]

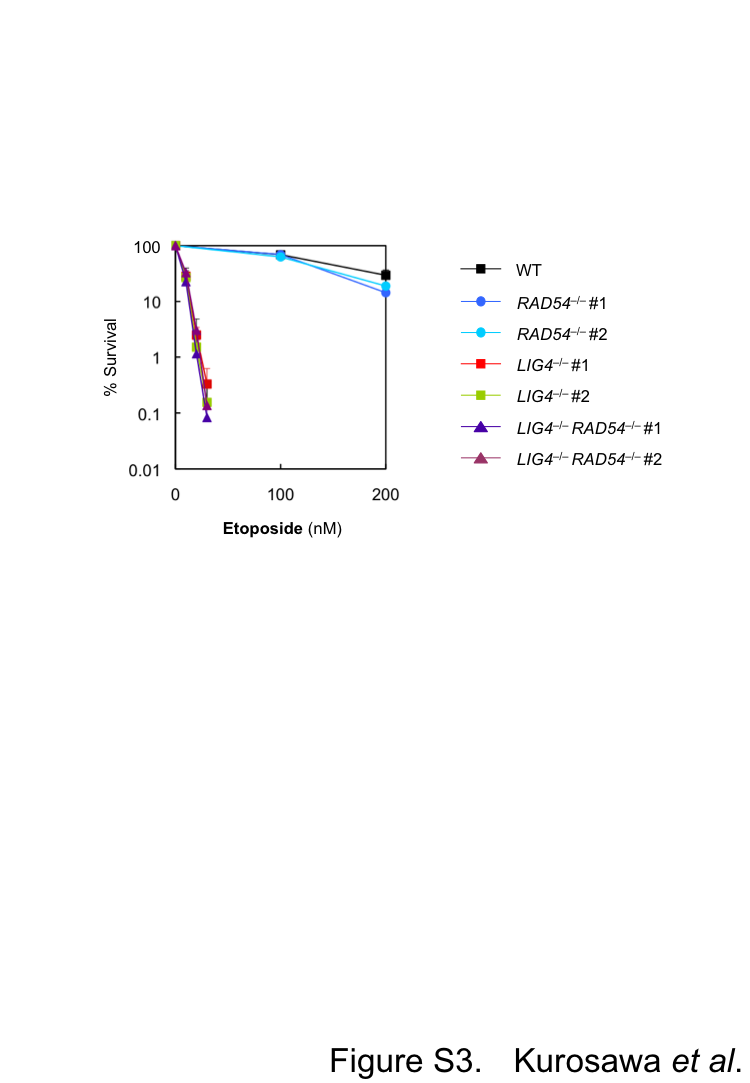

Supplement: Figure S3 — Absolute requirement of NHEJ in repair of etoposide-induced DNA damage. Etoposide sensitivity of various mutant cell lines was determined by clonogenic assays. Data are the mean ± SD of at least three independent experiments. Where absent, error bars fall within symbols. (TIF) [file pone.0072253.s003.tif]

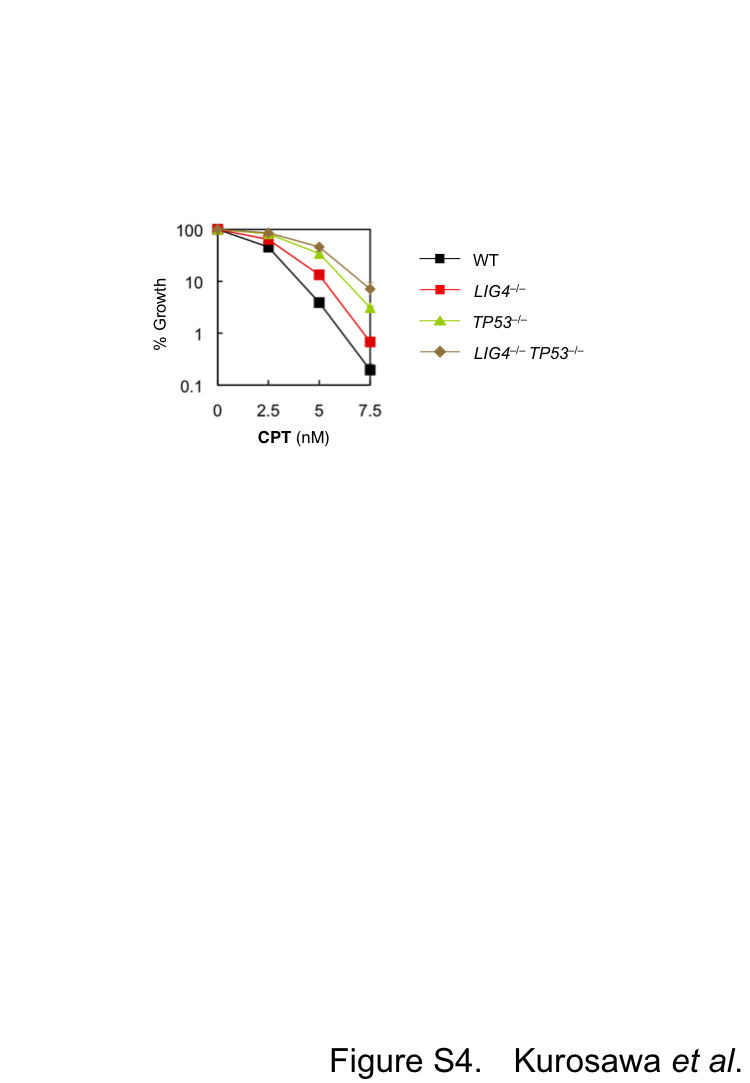

Supplement: Figure S4 — Increased resistance of LIG4 −/− cells to CPT is unrelated to p53 function. Shown is the sensitivity to CPT of wild-type (WT), LIG4 −/−, TP53 −/−, and LIG4 −/− TP53 −/− cells, as determined by growth inhibition assays. (TIF) [file pone.0072253.s004.tif]

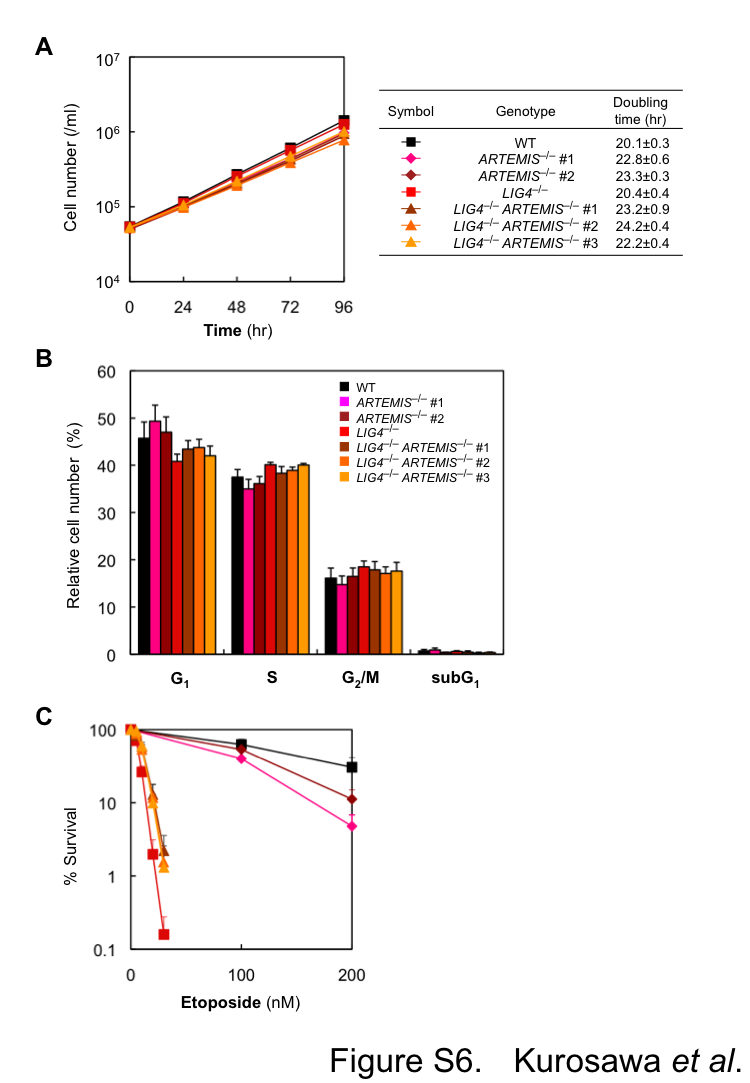

Supplement: Figure S6 — Growth properties of wild-type and mutant cell lines. (A) Growth curves of wild-type (WT), ARTEMIS −/−, LIG4 −/−, and LIG4 −/− ARTEMIS −/− cells. Shown are the mean ± SD of three independent experiments. (B) Percentage of cells in G1, S, G2/M, and subG1. Shown are the mean ± SD of three independent experiments. (TIF) [file pone.0072253.s006.tif]

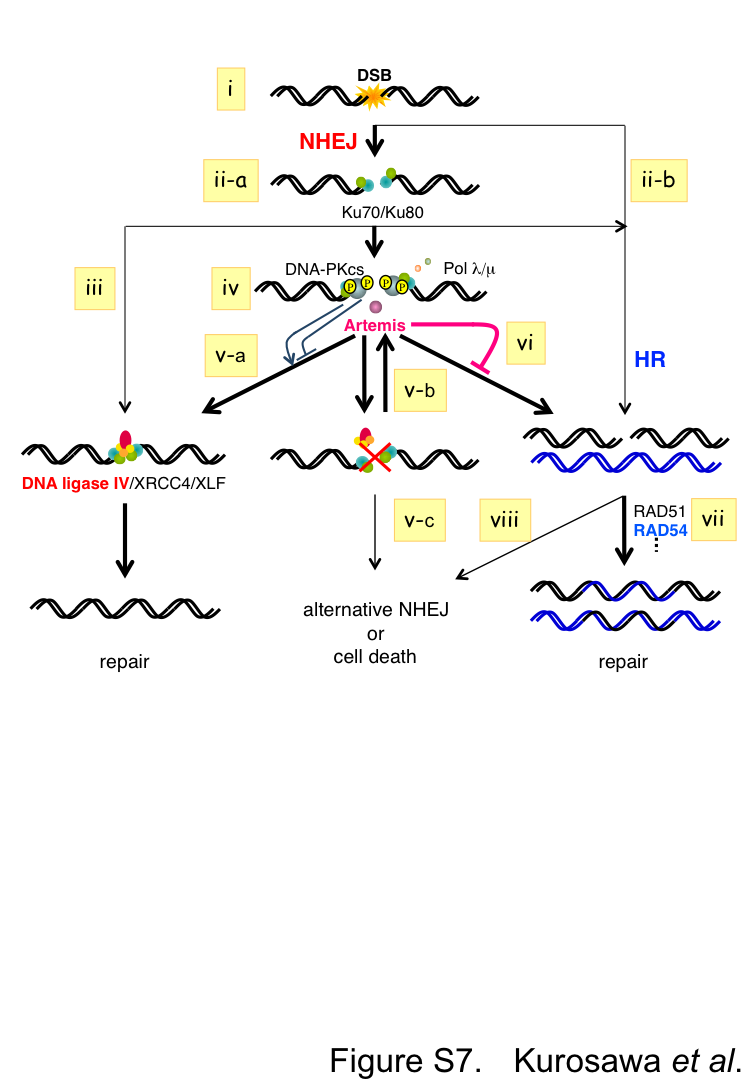

Supplement: Figure S7 — Model for DSB repair control in human somatic cells. Cells can suffer various types of DSBs, involving those induced by irradiation, Top2 and, in S phase, replication fork collapse (i). In this model, the Ku70/Ku80 complex can rapidly bind to most, if not all, DSBs to initiate an NHEJ reaction (ii-a). Ku-unbound DSBs, if any, are not repaired by NHEJ, but by HR (ii-b). After the Ku binding, the DNA ligase IV/XRCC4 complex rejoins the DSB when end-trimming is unnecessary (this would only be true for “clean” ends; e.g., signal joint formation during V(D)J recombination [5]) (iii). In most cases, end-trimming is required prior to rejoining, so that DNA-PKcs, Artemis, and DNA polymerases are recruited to the DSB to trim the ends (iv) [5]. After the trimming, the DSB can be rejoined by the DNA ligase IV/XRCC4 complex (v-a), in the absence of which, however, the break remains unrejoined [18]. Such situations can be caused by incomplete end-trimming reactions [61] and/or when the cell has a very large number of DSBs. (Regarding the latter, it is particularly interesting to note that the expression level of DNA ligase IV (and XRCC4) is considerably lower than Ku70/Ku80 [12]; thus, it is reasonable to speculate that all the Ku-bound DSBs cannot be rejoined by DNA ligase IV.) In these cases NHEJ may repeatedly perform end-trimming and ligation reactions (v-b). It could be that the presence of Artemis may assure these reactions; namely, Artemis may serve to suppress switching from the incomplete NHEJ reaction to HR (vi), though such unrejoined DSBs may cause cell death (v-c). When NHEJ is unsuccessful at rejoining the DSB, the cell gives up the abortive NHEJ reaction by somehow relieving the Artemis-mediated HR suppression. Then, HR finally gets the opportunity to repair the DSB (vii). (Possibly, DNA-PKcs may change its autophosphorylation status to facilitate HR [62].) Alternatively, or additionally, those DSBs that remain unrejoined may be shunted to an alternative end-joini [file pone.0072253.s007.tif]
